# International consensus on the prevention of venous and arterial thrombotic events in patients with inflammatory bowel disease

**DOI:** 10.1038/s41575-021-00492-8

**Published:** 2021-08-27

**Authors:** Pablo A. Olivera, Stephane Zuily, Paulo G. Kotze, Veronique Regnault, Sameer Al Awadhi, Peter Bossuyt, Richard B. Gearry, Subrata Ghosh, Taku Kobayashi, Patrick Lacolley, Edouard Louis, Fernando Magro, Siew C. Ng, Alfredo Papa, Tim Raine, Fabio V. Teixeira, David T. Rubin, Silvio Danese, Laurent Peyrin-Biroulet

**Affiliations:** 1 Gastroenterology Section, Department of Internal Medicine, Centro de Educación Médica e Investigaciones Clínicas (CEMIC), Buenos Aires, Argentina; 2 Vascular Medicine Division and Regional Competence Center for Rare Vascular and Systemic Autoimmune Diseases, Centre Hospitalier Régional Universitaire de Nancy, Vandoeuvre-lès-Nancy, France; 3 University of Lorraine, INSERM, DCAC, Nancy, France; 4 IBD outpatient clinics, Colorectal Surgery Unit, Catholic University of Paraná (PUCPR), Curitiba, Brazil; 5 Gastroenterology Division, Rashid Hospital, Dubai Health Authority, Dubai, UAE; 6 Imelda GI Clinical Research Center, Imelda General Hospital, Bonheiden, Belgium; 7 Department of Medicine, University of Otago, Christchurch, New Zealand; 8 NIHR Biomedical Research Centre, University of Birmingham and University Hospitals NHS Foundation Trust, Birmingham, UK; 9 Center for Advanced IBD Research and Treatment, Kitasato University, Kitasato Institute Hospital, Tokyo, Japan; 10 Department of Gastroenterology, CHU Liège University Hospital, Liège, Belgium; 11 Department of Gastroenterology, Centro Hospitalar São João, Porto, Portugal; 12 Department of Medicine and Therapeutics, Institute of Digestive Disease, LKS Institute of Health Science, The Chinese University of Hong Kong, Hong Kong SAR, China; 13 Division of Internal Medicine and Gastroenterology, Fondazione Policlinico Universitario “A. Gemelli” IRCCS, Rome, Italy; 14 Catholic University of Rome, Rome, Italy; 15 Department of Gastroenterology, Cambridge University Hospitals NHS Foundation Trust, Cambridge, UK; 16 Gastrosaúde - IBD Clinic, Marília, SP Brazil; 17 University of Chicago Medicine, Inflammatory Bowel Disease Center, Chicago, IL USA; 18 IBD Center, Department of Gastroenterology, Humanitas Clinical and Research Center - IRCCS, Milan, Italy; 19 Department of Biomedical Sciences, Humanitas University, Pieve Emanuele, Milan, Italy; 20 Department of Gastroenterology and INSERM NGERE U1256, University Hospital of Nancy, University of Lorraine, Vandoeuvre-lès-Nancy, France

**Keywords:** Inflammatory bowel disease, Cardiovascular diseases

## Abstract

Patients with inflammatory bowel disease (IBD) are at increased risk of thrombotic events. Therapies for IBD have the potential to modulate this risk. The aims of this Evidence-Based Guideline were to summarize available evidence and to provide practical recommendations regarding epidemiological aspects, prevention and drug-related risks of venous and arterial thrombotic events in patients with IBD. A virtual meeting took place in May 2020 involving 14 international IBD experts and 3 thrombosis experts from 12 countries. Proposed statements were voted upon in an anonymous manner. Agreement was defined as at least 75% of participants voting as ‘fully agree’ or ‘mostly agree’ with each statement. For each statement, the level of evidence was graded according to the Scottish Intercollegiate Guidelines Network (SIGN) grading system. Consensus was reached for 19 statements. Patients with IBD harbour an increased risk of venous and arterial thrombotic events. Thromboprophylaxis is indicated during hospitalization of any cause in patients with IBD. Disease activity is a modifiable risk factor in patients with IBD, and physicians should aim to achieve deep remission to reduce the risk. Exposure to steroids should be limited. Antitumour necrosis factor agents might be associated with a reduced risk of thrombotic events.

## Abstract (web summary)

Patients with inflammatory bowel disease (IBD) are at increased risk of thrombotic events. This Evidence-Based Guideline presents an international consensus on the prevention of venous and arterial thrombotic events in patients with IBD, and includes 19 recommendations for clinical practice.

## Introduction

**Fig. 1:**
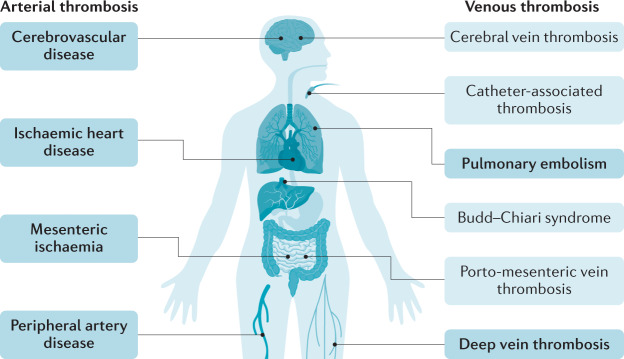
Arterial and venous sites of thrombosis in patients with IBD. Common sites of arterial and venous thrombosis are shown in bold, atypical sites are not bolded. IBD, inflammatory bowel disease.

Inflammatory bowel disease (IBD), namely Crohn’s disease and ulcerative colitis, is a group of systemic conditions with predominant intestinal inflammation^1,2^. Similar to other immune-mediated inflammatory diseases (IMIDs), such as psoriasis and rheumatoid arthritis, IBD can be associated with different comorbidities, including thrombosis^3^. Patients with IBD are known to carry an increased risk of developing both arterial and venous thrombosis^4^ (Fig. 1).

**Fig. 2:**
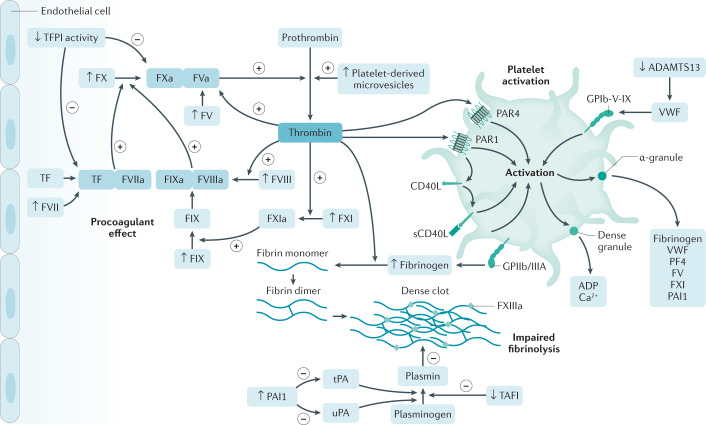
Pathophysiology of thrombosis in IBD. This figure depicts the alterations involved in the increased risk of thrombosis in patients with inflammatory bowel disease (IBD): platelet alterations leading to activation; procoagulant alterations leading to activation of the coagulation cascade; dysregulated fibrinolysis. ADAMTS13, a disintegrin and metalloproteinase with a thrombospondin type 1 motif, member 13; GPIIb/GPIIIA, glycoprotein IIb/IIIa; PAI1, plasminogen activator inhibitor 1; PAR, protease-activated receptor; PF4, platelet factor 4; TAFI, thrombin-activatable fibrinolysis inhibitor; TF, tissue factor; TFPI, tissue factor pathway inhibitor; tPA, tissue plasminogen activator; uPA, urokinase-type plasminogen activator; VWF, von Willebrand factor. Elements of Fig. 2 adapted with permission from ref.^7^, Elsevier.

Patients with IBD have several factors that influence the pathophysiological mechanisms of venous thromboembolism (VTE) encompassed in the well-known Virchow’s triad: stasis of venous flow (for example, bed rest), endothelial injury (for example, surgery or trauma) and hypercoagulability (for example, inflammation through several mechanisms, sepsis and antiphospholipid antibodies)^5–7^. Inflammation plays an important role in the development of accelerated atherosclerotic disease, which can lead to arterial thrombotic events^8^ (Fig. 2). This feature has been shown across the spectrum of IMIDs, as well as in the general population^9,10^.

Anti-inflammatory drugs used for the treatment of IBD can reduce inflammation and potentially the risk of thrombosis. Whether JAK inhibitors might have an intrinsic pro-thrombotic effect is the subject of intensive research^11^.

The aim of this Evidence-Based Guideline is to summarize available evidence and to provide practical recommendations agreed by consensus regarding epidemiological aspects, prevention, and drug-related risk of venous and arterial thrombotic events in patients with IBD.

## Methods

A literature search was conducted by P.A.O. and L.P.-B. to evaluate the current knowledge on the epidemiology of thrombotic events in IBD, and how available IBD therapies impact the risk of these events. Published studies were identified using MEDLINE and EMBASE from inception until 20 May 2020. Databases of major congresses (European Crohn’s and Colitis Organization, Digestive Disease Week, and United European Gastroenterology Week) in the period 2012–2019 were also reviewed manually. Search strategies are provided in Supplementary Table 1. Proposed statements were prepared by P.A.O. and L.P.-B. prior to the meeting and presented according to two predefined categories: epidemiology of thrombosis in IBD, and prevention of thrombosis in IBD, including drug-related. A consensus meeting was originally planned to take place in Lisbon, Portugal, on 27 May 2020. Owing to the COVID-19 pandemic, the consensus meeting took place virtually via the Zoom platform on the same day instead.

The objectives of the consensus were: to define the risk of both venous and arterial thrombotic events in patients with IBD; to give recommendations on reducing the risk of thrombotic events; to define the risk of drug-related thrombosis in patients with IBD; and to give recommendations on reducing the risk of drug-related thrombosis. Attendees of the virtual consensus included 14 IBD experts, three experts in clinical and basic aspects of thrombosis and one fellow, from 14 countries. During the meeting, the results of the literature review were presented, followed by presentation of the proposed statements. Each proposed statement was voted upon in an anonymous manner using the voting system of the Zoom platform. Agreement was defined as at least 75% of participants voting as ‘fully agree’ or ‘mostly agree’ with each proposed statement. If a 75% agreement was not achieved, further discussion ensued, which might have included amendment of voting statements when required, followed by a second round of voting using the same approach as before if the statement remained controversial. If agreement could not be reached after two rounds of voting, then the statement was definitely excluded. ﻿For each statement, the level of evidence was graded according to the Scottish Intercollegiate Guidelines Network (SIGN) grading system (Supplementary Table 2). The SIGN grading system is a widely used critical appraisal and evidence hierarchy that has the important advantage of being simple and easy to use^12^. The methodology is based on a set of variables that recognize key factors, especially bias and confounding factors, that can influence the quality of a study. The final grade of recommendation (A, B, C or D) is based on the lowest level of evidence applicable to a key outcome^12^. Consensus was reached for 19 statements (Box 1), and 6 statements were excluded (Supplementary Table 3).

Box 1 Consensus statements

- **Statement 1:** Inflammatory bowel disease (IBD) is associated with a twofold greater risk of venous thromboembolism (VTE) events (consensus reached for 94%). Evidence level 2+.
- **Statement 2**: Patients with IBD should be screened for VTE risk factors (consensus reached for 100%). Evidence level 4.
- **Statement 3**: The risk of VTE in patients with IBD is related to disease activity and it is further increased during hospitalization (consensus reached for 100%). Evidence level 2−.
- **Statement 4**: Thromboprophylaxis should be given to patients with IBD during hospitalization of any cause. Low molecular weight heparin or fondaparinux is recommended over low-dose unfractionated heparin. Prophylaxis should be maintained during the inpatient period. Extended duration of prophylaxis after discharge should be considered only in patients with strong risk factors for VTE (consensus reached for 89%). Evidence level 2+.
- **Statement 5**: Thromboprophylaxis should be considered in ambulatory patients with active IBD with known risk factors for VTE and maintained until the patient is in remission (consensus reached for 95%). Evidence level 4.
- **Statement 6**: Thromboprophylaxis does not increase the risk of further IBD-related gastrointestinal bleeding in patients with active disease (consensus reached for 100%). Evidence level 2+.
- **Statement 7**: IBD is associated with a small but significant increased risk of arterial thrombotic events, especially in young patients with active disease (consensus reached for 100%). Evidence level 2++.
- **Statement 8**: The risk of cerebrovascular accidents and ischaemic heart disease seems to be increased in female patients with IBD, but not in male patients (consensus reached for 94%). Evidence level 2++.
- **Statement 9**: The risk of peripheral artery disease is modestly increased, especially in young patients with Crohn’s disease (consensus reached for 87%). Evidence level 2+.
- **Statement 10**: The risk of mesenteric ischaemia is increased in patients with IBD, especially in young patients with ulcerative colitis (consensus reached for 93%). Evidence level 2+.
- **Statement 11**: Control of disease activity is an important factor in reducing the risk of venous and arterial thrombotic events in patients with IBD (consensus reached for 93%). Evidence level 4.
- **Statement 12**: Established cardiovascular disease risk factors should be actively investigated and controlled in patients with IBD (consensus reached for 100%). Evidence level 4.
- **Statement 13**: There is limited evidence regarding the effect on the risk of VTE of 5-aminosalicylic acid (5-ASA), thiopurines or methotrexate in patients with IBD (consensus reached for 100%). Evidence level 3.
- **Statement 14**: 5-ASA is associated with a reduced risk of ischaemic heart disease in patients with IBD, especially when given in the long term (consensus reached for 100%). Evidence level 2−.
- **Statement 15**: Steroids are associated with an increased risk of venous and arterial thrombotic events in patients with IBD (consensus reached for 100%). Evidence level 2++.
- **Statement 16**: Anti-TNF agents can be associated with a decreased risk of VTE in patients with IBD (consensus reached for 100%). Evidence level 2+.
- **Statement 17**: Anti-TNF agents can be associated with a reduced risk of arterial events in patients with IBD (consensus reached for 94%). Evidence level 2+.
- **Statement 18**: Tofacitinib can be associated with a dose-dependent increased risk of VTE in patients with rheumatoid arthritis with risk factors for VTE. According to available evidence, no increase in the risk of VTE has been observed in the overall ulcerative colitis population treated with tofacitinib (consensus reached for 100%). Evidence level 1+.
- **Statement 19**: Tofacitinib is not associated with an increased risk of major adverse cardiovascular events in patients with ulcerative colitis (consensus reached for 100%). Evidence level 1−.

## Risk of venous and arterial thrombotic events in IBD

### Statement 1: IBD is associated with a twofold greater risk of VTE events

- Consensus reached for 94%. Vote: fully agree 65%, mostly agree 29%.
- Evidence level 2+.

Several population-based IBD cohort studies have investigated the risk of VTE events^13–17^. A meta-analysis by Yuhara et al. summarizing data from 11 observational studies, found a relative risk (RR) of 2.20 (95% CI 1.83–2.65) for deep vein thrombosis (DVT) and pulmonary embolism (PE) in patients with IBD compared with individuals without IBD^18^. However, significant heterogeneity between studies was detected. An increased risk was seen in patients with ulcerative colitis (RR 2.57, 95% CI 2.02–3.28) and Crohn’s disease (RR 2.12, 95% CI 1.40–3.20)^18^. A separate meta-analysis by Fumery et al. that included ten studies involving both hospitalized and ambulatory patients, found an increased risk of VTE in patients with IBD, with a RR of 1.96 (95% CI 1.67–2.30)^19^. The risk in patients with Crohn’s disease and ulcerative colitis did not differ, except when considering only studies evaluating hospitalized patients, in whom the increased risk of VTE was greater in those with ulcerative colitis (*P* = 0.0029)^19^.

The risk of VTE can be further modulated by the presence of other factors frequently seen in patients with IBD, such as surgery, pregnancy, disease activity and hospitalizations (see Statement 3 and Table 1). Additionally, other risk factors should also be taken into account (see Statement 2).

**Table 1:** Risk of VTE in different clinical scenarios

Setting	Findings	Ref.
Hospitalization	The incidence rate of VTE in patients with active IBD flare during hospitalization was the highest (37.5 per 1,000 patient-years) The relative risk when compared with controls increased threefold (HR 3.2, 95% CI 1.7–6.3)	Grainge et al.^51^
	Among hospitalized patients, those with ulcerative colitis (OR 1.85, 95% CI 1.70–2.01) and those with Crohn’s disease (OR 1.48, 95% CI 1.35–1.62) had higher rates of VTE than patients without IBD VTE was associated with longer hospital stays, higher hospitalization-related costs and higher mortality among patients with IBD (adjusted OR 2.50, 95% CI 1.83–3.43)	Nguyen and Sam^15^
	IBD-related surgery (adjusted HR 40.81; 95% CI 10.16–163.92) and hospitalization due to flare-up (adjusted HR 19.36, 95% CI 9.59–39.07) were the highest risk factors for VTE The risk of VTE also remained elevated in patients hospitalized without flare-up (adjusted HR 12.97, 95% CI 8.68–19.39)	Kim et al.^54^
	IBD-related hospitalization was the strongest risk factor for VTE (OR 1.72, 95% CI 1.39–2.12)	Ananthakrishnan et al.^56^
Early discharge	Thromboprophylaxis during the index hospitalization was associated with a significantly lower risk of post-hospitalization VTE (HR 0.46, 95% CI 0.22–0.97)	Ananthakrishnan et al.^56^
	91% of readmissions due to VTE after an index IBD hospitalization occurred in the 60 days following discharge, with the risk being highest in the first 10 days after discharge	Faye et al.^68^
	30-day total and post-discharge rates of VTE were 2.5% and 1% following elective abdominopelvic bowel surgery in patients with IBD	Benlice et al.^49^
	The risk of VTE remained elevated within 6 weeks of discharge in patients with IBD following major surgery	Chu et al.^69^
Ambulatory	The relative risk during flare in the ambulatory setting was higher (HR 15.8, 95% CI 9.8–25.5; *P* < 0.0001) than during hospitalizations (HR 3.2, 95% CI 1.7–6.3), when compared with the risk in the general population	Grainge et al.^51^
	Most VTE events (77.8%) occurred in outpatients	Papay et al.^72^

IBD, inflammatory bowel disease; VTE, venous thromboembolism.

### Statement 2: Patients with IBD should be screened for VTE risk factors

- Consensus reached for 100%. Vote: fully agree 75%, mostly agree 25%.
- Evidence level 4.

Several inherited and acquired factors are related with an increased risk of VTE. Patients with IBD should be thoroughly investigated for additional risk factors of thrombosis, as the presence of multiple factors might increase the risk further^20^. The overall risk assessment should be taken into account when making clinical decisions.

VTE events are categorized as provoked and unprovoked, depending on the presence of an acquired risk factor^21^ (Box 2). Additionally, these factors can be transient (for example, surgery) or persistent (for example, antiphospholipid syndrome), which could have potential implications on prognosis and treatment decisions^21^. When the VTE event is provoked by a transient major risk factor, the risk of recurrent VTE is low after stopping therapy when the risk factor has disappeared, and long-time anticoagulation is not warranted in general^22^. In case of a persistent major risk factor, such as active cancer, the risk of recurrence is high as long as the factor is present and, therefore, anticoagulation should be continued.

Patients with IBD do not seem to be at an increased risk of inherited thrombophilia^23,24^. Several studies have shown that the prevalence of genetic mutations associated with an increased risk of VTE (for example, factor V Leiden polymorphism^25,26^, G20210A mutation in the prothrombin gene^27^, and homozygous C677T mutation in the *MTHFR* gene^24,28–30^) are similar in patients with and without IBD, and also when comparing patients with IBD with or without VTE^23,31–40^. Consequently, patients with IBD should not be routinely screened for genetic or acquired laboratory abnormalities related to thrombophilia, with the exception of the occurrence of an unprovoked VTE event in the absence of risk factors (Box 2) and intestinal inflammation (that is, patient in deep remission).

Acquired risk factors for VTE include a history of a VTE event, older age (>65 years), obesity, ongoing malignancy and recent major surgery (higher after orthopaedic surgery), as well as other medical conditions^41^ (Box 2). These clinical risk factors should be assessed in all patients with IBD, according to existing guidelines at baseline^42^. Many of these risk factors are transient, and need a continuous re-evaluation, especially when the clinical condition changes (such as flare, hospitalization, surgery, complication, discharge and so on). Different thrombosis risk assessment models (RAMs) have been developed for specific clinical scenarios^43^. Notably, most of these RAMs do not include IBD as a risk factor. A widely used model is the modified Caprini RAM for use in patients undergoing surgery^44^. For non-surgical inpatients who are acutely ill, several RAMs have been developed (such as the Padua prediction score^45^, the IMPROVE risk score^46^ and the GENEVA risk score^47^), although their performance is variable. Another example is the Khorona score, which predicts the risk of VTE in ambulatory patients with cancer^48^. Unfortunately, there is a paucity of specific and validated screening tools for the assessment of the overall risk of VTE in patients with IBD. Two RAMs were developed for use in the IBD population, one to predict the risk of VTE after abdominal surgery^49^, and the other to predict the risk of VTE after discharge^50^ (see Statement 4), but neither has been validated. Development of validated tools is imperative to enable correct assessment of the risk of thrombosis and to adequately implement tailored prophylactic measures in different clinical scenarios.

Box 2 Risk factors for venous thrombotic events **Major risk factors**

- Active malignancy
- Recent (within 3 months) surgery with general anaesthesia for >30 min
- Trauma of lower limbs
- High-risk thrombophilia (for example, antiphospholipid syndrome, antithrombin deficiency)
- Immobilization (confinement to bed in hospital with bathroom privileges for an acute medical condition for >3 days)

**Minor risk factors**

- Recent (within 3 months) surgery with general anaesthesia for <30 min
- Venous catheter
- Older age (>65 years)
- Pregnancy and post-partum period (2 months after delivery)
- Oral contraceptives containing oestrogens
- Hormone-replacement therapy
- Lower-risk thrombophilia (for example, factor V Leiden polymorphism, prothrombin gene mutation)
- History of venous thromboembolism
- Hyperhomocysteinaemia
- Obesity
- Long-haul flights

### Statement 3: The risk of VTE in patients with IBD is related to disease activity and it is further increased during hospitalization

- Consensus reached for 100%. Vote: fully agree 82%, mostly agree 18%.
- Evidence level 2−.

Disease activity has an important role in the risk of VTE in patients with IBD. Additionally, this risk seems to be modulated by the setting of the IBD flare (hospitalization, early discharge or ambulatory (Table 2, see Statements 4 and 5)).

**Table 2:** Core recommendations

Recommendation	Strength of recommendation^a^	Corresponding statement
Patients with IBD should be screened for VTE risk factors (note: patients should not be routinely screened for genetic inherited thrombophilia)	D	Statement 2
Thromboprophylaxis should be given to patients with IBD during hospitalization of any cause and maintained during the inpatient period	C	Statement 4
Extended-duration prophylaxis after discharge should be considered only in patients with strong risk factors for VTE	D	Statement 4
Thromboprophylaxis should be considered in ambulatory patients with active IBD with known risk factors for VTE	D	Statement 5
Disease activity should be resolved to reduce the risk of thrombotic events; deep remission should be the target	D	Statement 11
Established cardiovascular disease risk factors should be actively investigated and controlled in patients with IBD	C	Statement 12
Smoking cessation should be encouraged	B	Statement 12
Folate supplementation should be advised in patients with IBD on methotrexate to avoid hyperhomocysteinaemia	C	Statement 13
Steroid exposure should be limited to prevent venous and arterial thrombotic events	B	Statement 15
Tofacitinib 10 mg BID should be used as induction therapy for up to 16 weeks; tofacitinib 5 mg BID should be the preferred maintenance dose; in patients with an insufficient response to the maintenance dose, dose increase to 10 mg BID could be considered in patients without known risk factors of VTE and without therapeutic alternatives	B	Statement 18

BID, twice daily; IBD, inflammatory bowel disease; VTE, venous thromboembolism. ^a^According to the SIGN grading system (Supplementary Table 2).

In a large cohort study from the UK (comparing 13,756 patients with IBD and 71,672 matched controls), Grainge et al. found that patients with IBD have a higher risk of VTE than controls (up to five controls matched for age, sex and general practice were selected for every patient with IBD from the General Practice Research Database) with a hazard ratio of 3.4 (95% CI 2.7–4.3)^51^. During periods of active flare, the risk was more prominent (HR 8.4, 95% CI 5.5–12.8) than during periods of remission (HR 2.1, 95% CI 1.6–2.9) compared with that in controls﻿. The incidence of VTE in patients with active IBD flare during hospitalization remained the highest. However, the relative risk during flare in the ambulatory setting was higher (HR 15.8, 95% CI 9.8–25.5; *P* < 0.0001) than during hospitalization (HR 3.2, 95% CI 1.7–6.3) compared with that in the general population^51^. Importantly, the definition of active disease in this study was corticosteroid use, so this study could not adequately separate the effects of active inflammation from the effects of the corticosteroids on VTE. Additionally, corticosteroids are usually indicated for the treatment of moderate-to-severe IBD disease activity. Hence, the influence of mild IBD compared with true remission remains unclear.

In a single-centre cohort study that included 84 patients with IBD, 71% of patients with IBD with a history of thrombotic events had active disease at the time of the event^52^. A population-based cohort study, which ﻿included 1,046,754 women with 1,978,701 deliveries, showed that disease flare-up during pregnancy was associated with a statistically significantly increased risk of VTE (﻿RR 2.64, 95% CI 1.69–4.14) when compared with the risk in pregnant patients with IBD without flare-ups during pregnancy^53^.

Nguyen and Sam found higher rates of VTE in hospitalized patients with IBD than in hospitalized patients without IBD. In this study, VTE was associated with longer hospital stays, higher hospitalization-related costs and, importantly, ﻿a greater mortality among patients with IBD (adjusted OR 2.50, 95% CI 1.83–3.43)^15^. In a population-based study from Korea, Kim et al. concluded that the risk of VTE was the highest during IBD-related surgery and hospitalization due to flare-up^54^. Importantly, the risk of VTE also remained elevated during hospitalization not due to flare up in another study^55^, highlighting the need for thromboprophylaxis in patients hospitalized for any cause (see Statement 4). In a retrospective multicentre cohort study that included 11,028 patients with IBD, of whom 2,788 had had at least one IBD-related hospitalization, Ananthakrishnan et al.^56^ found that IBD-related hospitalization was the strongest risk factor for VTE (OR 1.72, 95% CI 1.39–2.12).

### Statement 4: Thromboprophylaxis should be given to patients with IBD during hospitalization of any cause. Low molecular weight heparin or fondaparinux is recommended over low-dose unfractionated heparin. Prophylaxis should be maintained during the inpatient period. Extended duration of prophylaxis after discharge should be considered only in patients with strong risk factors for VTE

- Consensus reached for 89%. Vote: fully agree 18%, mostly agree 71%.
- Evidence level 2+.

In accordance with current guidelines on the prevention of VTE during hospitalization, patients with IBD should receive pharmacological thromboprophylaxis during the inpatient period regardless of the cause of hospitalization^32,42,57^. In the study by Grainge et al., hospitalized patients with IBD had higher risk of VTE than patients without IBD, even during periods of remission (HR ﻿1.7, 95% CI 1.1–2.9; *P* = 0.03)^51^, implying that patients with IBD might benefit from thromboprophylaxis during hospitalization of any cause. In another study, ﻿thromboprophylaxis during the index hospitalization was associated with a significantly lower risk of VTE after discharge (HR 0.46, 95% CI 0.22–0.97)^56^.

Guidelines recommend pharmacological over mechanical thromboprophylaxis, given that the former may be more effective in the prevention of PE and symptomatic VTE^32,42,58,59^. The preferred methods of prophylaxis are low molecular weight heparin (LMWH) or fondaparinux over unfractionated heparin (UFH)^42^. ﻿LMWH was associated with lower rates of PE, symptomatic DVT, major bleeding and heparin-induced thrombocytopenia than UFH^42,60^. Additionally, fondaparinux might reduce mortality, PE, distal DVT and major bleeding, when indirectly compared with UFH and LMWH^42^. A combined analysis of three randomized controlled trials (RCTs) that evaluated direct oral anticoagulants (DOACs) against LMWH for primary prevention of VTE in medical inpatients^61–63^ showed that DOACs are not associated with a clinical benefit compared with LMWH and led to an increased risk of major bleeding^42^. Thus, DOACs are not recommended as primary prophylaxis of VTE during hospitalization in all patients, including those with IBD.

An extended duration of thromboprophylaxis beyond hospital discharge is not routinely recommended in patients with IBD. However, surgical and orthopaedic research have shown that the risk of VTE can remain elevated even after hospital discharge^64–66^, and a subgroup of patients might benefit from an extended duration of VTE prophylaxis^67^. In a retrospective cohort study that analysed ﻿872,122 index admissions of patients with IBD, 91% of readmissions due to VTE occurred in the 60 days following discharge, with the risk being highest in the first 10 days after discharge^68^. ﻿Factors associated with readmission with VTE included older age, presence of comorbidities, history of VTE, ﻿having a flexible sigmoidoscopy or colonoscopy on index admission, *Clostridioides difficile* infection, discharge to a nursing or intermediate facility and discharge home with services^68^. In a large retrospective cohort study that included ﻿24,182 patients, 30-day total and post-discharge rates of VTE were 2.5% and 1%, respectively, following elective ﻿abdominopelvic bowel surgery in patients with IBD^49^. Factors associated with increased rates of post-discharge VTE were preoperative transfusion, steroid use, pelvic and enterocutaneous fistula surgery, and longer operative time^49^. Additionally, a study by Chu et al. also found that the risk of VTE remained elevated within 6 weeks of discharge in patients with IBD following major surgery^69^. The American Society of Colon and Rectal Surgeons clinical practice guidelines do recommend extended thromboprophylaxis in selected high-risk patients with IBD following abdominal surgery (weak recommendation, very low quality evidence)^59^. The cost-effectiveness of this strategy remains to be fully defined. A Canadian study showed that extended thromboprophylaxis (28-day course of enoxaparin) after colorectal surgery due to malignancy or IBD had higher costs than standard management (inpatient administration only), but more quality-adjusted life-years (QALYs), and an incremental cost-effectiveness ratio^70^. However, in a study by Leeds et al., extended prophylaxis following surgery for Crohn’s disease was not cost-effective when the cumulative incidence of VTE after hospitalization remained <4.9%^71^.

Consequently, an extended duration of thromboprophylaxis can be considered for an additional period of 2–8 weeks after hospital discharge in patients with a very high risk of VTE, and identification of these patients is of the utmost importance. In a single-centre retrospective study published in 2019, McCurdy et al. developed a risk model for VTE after discharge that included age >45 years, multiple admissions, intensive care unit admission, length of admission >7 days and presence of a central catheter; by limiting extended thromboprophylaxis to high-risk patients identified by the score, the authors concluded that treatment could be avoided in 92% of patients after discharge^50^. However, this RAM needs further validation, and until then physicians should rely on clinical gestalt to estimate the risk of VTE after hospital discharge.

### Statement 5: Thromboprophylaxis should be considered in ambulatory patients with active IBD with known risk factors for VTE, and maintained until the patient is in remission

- Consensus reached for 95%. Vote: fully agree 24%, mostly agree 71%.
- Evidence level 4.

Moderate to severe IBD activity is a known risk factor for VTE. Most IBD flares occur in an ambulatory setting but given that the thresholds for hospitalization due to IBD flare differ worldwide, the proportions of inpatients and outpatients with IBD having a VTE event can vary. Moderate to severe IBD should be considered as a risk factor for VTE in the presence of clinical signs of activity in addition to objective markers of inflammation (that is, elevated C-reactive protein (CRP) or calprotectin levels, or signs of moderate to severe inflammation in endoscopic or cross-sectional evaluations).

In the study by Grainge et al., the relative risk of VTE during a disease flare compared with that in the general population was higher during ambulatory periods (HR 15.8, ﻿95% CI 9.8–25.5) than during hospitalized periods (HR 3.2, 95% CI 1.7–6.3)^51^. An Austrian multicentre cohort study that included ﻿2,784 patients with IBD (total observation time 24,778 person-years) found that most VTE events (77.8%) occurred in outpatients^72^. Additionally, 77.1% of VTE events were unprovoked by classic provoking risk factors, such as surgery, trauma or indwelling catheters, although almost two-thirds (60.9%) of the patients had active disease at the time of first VTE^72^. However, in the study by Grainge et al., the incidence of VTE during flares was much lower during ambulatory periods than during hospitalized periods (6.4 per 1,000 person-years versus 37.5 per 1,000 person-years)^51^. Given that the absolute risk of VTE remains low in outpatients with active IBD, the use of thromboprophylaxis is not recommended in the absence of risk factors. In a Markov decision analysis, it was estimated that thromboprophylaxis during ambulatory IBD flares was not cost-effective, ﻿with a number needed to treat to prevent one VTE event over a lifetime of 32.3 and a cost of US$1,267,450 for every QALY gained^73^.

On the other hand, outpatients with major risk factors or multiple risk factors in addition to a moderate to severe IBD flare might be at a particular high risk of VTE (Box 2) and could benefit from thromboprophylaxis until the transient provoking factor disappears (that is, patient achieves remission). This approach might be particularly relevant in patients with a history of VTE provoked by IBD flare who are no longer on anticoagulation. A cohort study by Novacek et al. found that after a first episode of VTE, patients with IBD had a higher risk of recurrent VTE than patients without IBD (HR 2.5, 95% CI 1.4–4.2; *P* = 0.001)^74^. There is a paucity of evidence regarding the efficacy and safety of thromboprophylaxis in patients with active IBD in the ambulatory setting, and this issue should be considered on a case-by-case basis.

### Statement 6: Thromboprophylaxis does not increase the risk of further IBD-related gastrointestinal bleeding in patients with active disease

- Consensus reached for 100%. Vote: fully agree 41%, mostly agree 59%.
- Evidence Level 2+.

Despite current recommendations, physicians are frequently concerned about the safety of thromboprophylaxis in patients with IBD, especially those presenting with overt gastrointestinal bleeding due to intestinal inflammation. This issue has been highlighted by a retrospective cohort study that included ﻿22,499 patients (474 with IBD), in which patients with IBD were less likely to receive thromboprophylaxis (79% with IBD versus 87% without IBD, *P < *0.01), particularly if haematochezia was present (OR 0.27, 95% CI 0.16–0.46)^75^. Accumulating evidence shows that thromboprophylaxis is safe in patients with IBD. A meta-analysis from 2007 evaluating the efficacy and safety of adjuvant heparin in active ulcerative colitis concluded that it was not associated with an increased risk of adverse events^76^. A retrospective study that included ﻿974 patients hospitalized for IBD showed that ﻿the incidences of major and minor bleeding were similar between hospitalized patients with IBD who did and did not receive thromboprophylaxis^77^; also VTE prophylaxis was not associated with major postoperative bleeding (0.4% versus 0%, *P* = 0.96)^77^. A single-centre retrospective study found that in ﻿233 patients with IBD hospitalized due to disease flare-up, thromboprophylaxis was associated with a lower odds of overall bleeding events (OR 0.19, 95% CI 0.14–0.26; *P* < 0.001), and there were no significant differences in haemoglobin levels compared with patients not on chemical thromboprophylaxis (*P* = 0.64)^78^. Importantly, in a study by Faye et al., ﻿thromboprophylaxis was not associated with increased blood transfusion requirements (14% versus 15%; *P* = 0.78) or with clinically significant decline in haemoglobin levels during hospitalization (﻿*P* = 0.25)^75^.

Nevertheless, prophylactic anticoagulation can be associated with further bleeding from other sites or even from the gastrointestinal tract in lesions unrelated to IBD (such as peptic ulcer disease, or bleeding tumours). The overall bleeding risk should be assessed prior to initiation of thromboprophylaxis using either clinical judgement or RAM. A validated RAM for this purpose is the IMPROVE bleeding risk score that helps identify acutely ill inpatients with an increased risk of bleeding^79–81^, in whom the risk–benefit ratio of a pharmacological thromboprophylaxis might be unfavourable. Notably, the IMPROVE score was developed in a general medical inpatient population and might not be necessarily be suitable for use in the IBD population. In patients with increased bleeding risk or contraindications to pharmacological thromboprophylaxis, mechanical prophylaxis (especially intermittent pneumatic compression) is preferred over no treatment^32,42,58^.

### Statement 7: IBD is associated with a small but significant increased risk of arterial thrombotic events, especially in young patients with active disease

- Consensus reached for 100%. Vote: fully agree 41%, mostly agree 59%.
- Evidence level 2++.

Atherosclerosis in several arterial beds can lead to thrombotic events, and systemic inflammation is a known risk factor for these events^82^. Patients with IBD have a slightly increased risk of arterial thrombotic events, even though traditional risk factors for cardiovascular atherosclerotic disease (CVD), such as diabetes, smoking, hyperlipidaemia, obesity and hypertension, are not universally over-represented in patients with IBD. In young patients, smoking, obesity and hypertension can explain most arterial thromboses because their attributable risks are high^83^. ﻿In a historical cohort study, ﻿Aggarwal et al. included 131 patients with IBD diagnosed with coronary artery disease (CAD) by ﻿cardiac catheterization and matched them with ﻿524 controls without IBD with CAD^84^. Patients with IBD were younger (65.3 ± 10.0 versus 67.8 ± 11.0 years; *P* = 0.016), had a lower prevalence of active smoking status (10.7% versus 18.7%; *P* = 0.03) and had a lower BMI (28.0 ± 5.1 kg/m^2^ versus 29.4 ± 6.4 kg/m^2^; *P* = 0.026) compared with controls^84^.

In a meta-analysis from 2014, Fumery et al. summarized data from nine observational studies evaluating the risk of ﻿arterial thrombotic events in patients with IBD, including stroke, peripheral artery disease (PAD), mesenteric ischaemia and ischaemic heart disease (IHD)^19^. They found an overall increased risk of these events in ambulatory patients with IBD (RR 1.28, 95% CI 1.16–1.42), although the difference did not reach statistical significance when including studies with both ambulatory and hospitalized patients ﻿(RR 1.15, 95% CI 0.91–1.45)^19^. In a population-based study from Copenhagen County that included ﻿108,789 participants (of whom 1,203 had IBD), Aarestrup et al. found that patients with IBD had a higher prevalence of CVD than individuals in the general population (13.2% versus 10.9%; *P* = 0.009)^85^. Importantly, ﻿traditional cardiovascular risk factors were not increased in patients with IBD and they actually had lower total and LDL cholesterol and blood pressure levels. On the other hand, patients with IBD had higher levels of CRP and fibrinogen as markers of chronic systemic inflammation, which could be the main driver of the increased risk of CVD in these patients^85^. In another Danish cohort study, Kristensen et al. found that ﻿patients with IBD had an overall increased risk of myocardial infarction (RR 1.17, 95% CI 1.05–1.31), stroke (RR 1.15, 95% CI 1.04–1.27) and cardiovascular death (RR 1.35, 95% CI 1.25–1.45)^86^. During periods of disease activity these risks increased even more^86^.

In a French population-based study, ﻿Kirchgesner et al. found a statistically significant increase in the risk of acute arterial thrombotic events (IHD, PAD and cerebrovascular disease; mesenteric ischaemia was excluded) compared with that in the general population (﻿standardized incidence rate (SIR) 1.19; 95% CI 1.16–1.22)^87^. ﻿The risk was higher in patients with Crohn’s disease than in patients with ulcerative colitis (SIR 1.35, 95% CI 1.30–1.41 versus SIR 1.10, 95% CI 1.06–1.13, respectively). Periods of active disease ﻿(defined as 3-month periods before and after IBD-related hospitalization or surgery) were independently associated with an increased risk of arterial events in both patients with Crohn’s disease ﻿(HR 1.74, 95% CI 1.44–2.09) and patients with ulcerative colitis (HR ﻿1.87, 95% CI 1.58–2.22)^87^. A nested case–control study from France also found that diabetes ﻿(OR 14.5, 95% CI 1.1–184.7) and clinical disease activity ﻿(OR 10.4, 95% CI 2.1–49.9) were independently associated with acute arterial thrombotic events in patients with IBD^88^.

A cohort study of ﻿31,175 patients with IBD and 154,412 matched controls without IBD found an increased hazard of myocardial infarction in ambulatory patients during periods of acute disease activity (HR 1.83, 95% CI 1.28–2.62) and chronic disease activity (HR 1.69, 95% CI 1.24–2.30)^89^. However, patients with IBD do not seem to have an increased risk of cardiovascular mortality. In a meta-analysis published in 2018, Sun and Tian found pooled standardized mortality ratios of 1.01 (95% CI 0.90–1.14) for patients with Crohn’s disease and 0.93 (95% CI 0.86–1.01) for patients with ulcerative colitis with low heterogeneity across studies^90^.

### Statement 8: The risk of cerebrovascular accidents and ischaemic heart disease seems to be increased in female patients with IBD, but not in male patients

- Consensus reached for 94%. Vote: fully agree 25%, mostly agree 69%.
- Evidence level 2++.

In a meta-analysis from 2014, Singh et al. found a slightly increased risk ﻿(adjusted OR 1.18, 95% CI 1.09–1.27) of cerebrovascular accident (CVA) in patients with IBD. Importantly, ﻿the risk of CVA was significantly higher in female patients (adjusted OR 1.28, 95% CI 1.17–1.41) but not in male patients (adjusted OR 1.11, 95% CI 0.98–1.25) with IBD (*P* = 0.05)^91^. Additionally, ﻿the magnitude of increased CVA risk was higher in young patients (<40–50 years) with IBD (adjusted OR 1.84, 95% CI 1.28–2.66) than in older patients with IBD (adjusted OR 1.11, 95% CI 1.02–1.21). Older patients usually have less severe disease with a milder course of IBD than younger patients; thus, the increased risk of these events seen in younger patients could be explained by higher disease activity. Also, with increasing age, traditional cardiovascular risk factors become more prevalent, which might outweigh the risk from milder IBD seen in the older population.

The meta-analysis by Fumery et al. did not find differences in the risk of IHD in patients with IBD ﻿(RR 1.23, 95% CI 0.94–1.62)^19^. However, in the meta-analysis by Singh et al., which included six studies, the risk of this event was slightly increased ﻿(adjusted OR 1.18, 95% CI 1.08–1.31)^91^. As seen with the risk of CVA, the increased risk of IHD in patients with IBD was seen in female patients (adjusted OR 1.26, 95% CI 1.18–1.35), but not in male patients (adjusted OR 1.05, 95% CI 0.92–1.21). Sex differences in the risk of CVA and IHD in patients with IBD might be explained by a greater role of systemic inflammation and hormonal differences in female patients^92^. Additionally, the higher background risk in male patients of these events might outweigh the increased risk attributed to IBD. The increased risk of IHD has also been shown in an updated meta-analysis by Feng et al. ﻿(adjusted RR 1.24, 95% CI 1.14–1.35)^93^. ﻿Again, in a subgroup analysis, the risk of IHD was more pronounced among female patients (adjusted RR 1.351, 95% CI 1.206–1.513) than among male patients (adjusted RR 1.189, 95% CI 1.028–1.375)^93^.

### Statement 9: The risk of peripheral artery disease is modestly increased, especially in young patients with Crohn’s disease

- Consensus reached for 87%. Vote: fully agree 40%, mostly agree 47%.
- Evidence level 2+.

Two meta-analyses did not show an increased risk of PAD in patients with IBD, but both meta-analyses included only two studies^19,91^. In a ﻿nationwide population-based cohort study from Taiwan (11,067 patients with IBD; 43,765 age, sex and comorbidity-matched controls), Lin et al. found that the risk of PAD was increased in patients with IBD (adjusted HR ﻿1.24, 95% CI 1.22–1.37; adjusted for age, sex and comorbidities)^94^. Interestingly, in patients with more than two ﻿annual IBD-related medical hospitalizations, the risk of developing PAD was the highest (adjusted HR ﻿27.5, 95% CI 18.7–40.4), showing a possible association with disease activity^94^.

In a more recent French cohort study by Kirchgesner et al., the risk of PAD was significantly increased in patients with IBD compared with the risk in the general population (SIR 1.27, 95% CI 1.17–1.37). This risk was significantly higher in patients with Crohn’s disease (SIR ﻿1.65, 95% CI 1.46–1.83), but not in those with ulcerative colitis ﻿(SIR 1.07, 95% CI 0.96–1.18)^87^. In patients with Crohn’s disease, the relative risk of PAD was the highest in those younger than 35 years (SIR 3.04, 95% CI 1.45–4.63) and this risk decreased successively with increasing age, becoming non-significant in those older than 75 years.

### Statement 10: The risk of mesenteric ischaemia is increased in patients with IBD, especially in young patients with ulcerative colitis

- Consensus reached for 93%. Vote: fully agree 33%, mostly agree 60%.
- Evidence level 2+.

Factors, such as local ﻿leukocyte infiltration and cytokine production, might interact with systemic inflammation, potentially leading to a particularly increased risk of this arterial event in patients with IBD^95,96^. The meta-analysis by Fumery et al. examined the risk of mesenteric ischaemia in patients with IBD. They included only two studies, and found a significant increased risk of this event in patients with IBD (﻿RR 3.46, 95% CI 1.78–6.71)^19^. In a large nationwide cohort study from Taiwan, which included 9,363 patients with IBD and 37,452 matched controls without IBD, the long-term risk of mesenteric ischaemia was significantly higher in patients with IBD than in controls, with the risk of mesenteric ischaemia within 13 years more than sixfold higher (adjusted HR ﻿6.33, 95% CI 4.75–8.43)^97^. The magnitude of the risk of mesenteric ischaemia also seems to be associated with younger age, with the risk almost 50-fold higher in patients with IBD younger than 45 years than in individuals without IBD (adjusted HR ﻿48.2, 95% CI 11.4–205.0). Additionally, patients with ulcerative colitis have approximately twice the risk of mesenteric ischaemia compared with patients with Crohn’s disease (adjusted HR ﻿2.07, 95% CI 1.41–3.03)^97^.

### Statement 11: Control of disease activity is an important factor in reducing the risk of venous and arterial thrombotic events in patients with IBD

- Consensus reached for 93%. Vote: fully agree 40%, mostly agree 53%.
- Evidence level 4.

Moderate to severe IBD activity has been identified as a risk factor for both VTE (see Statements 3 and 5) and arterial thrombotic events (see Statement 7). Disease activity should be regarded as a modifiable risk factor for these events, and aggressive control of inflammation might reduce the risk of thrombosis in patients with IBD. Physicians should aim for combined clinical and endoscopic remission, given that persistent subclinical inflammation can also increase the risk of events. In the general population, elevation of inflammatory markers, particularly CRP, is associated with an increased risk of IHD^9^. IBD therapies have the potential to reduce these risks by halting inflammation and, therefore, to reduce the risk of thrombotic events. However, some IBD therapies might have an intrinsic pro-thrombotic effect that could tilt the balance towards an increased risk of venous and/or arterial events (see Statements 13 to 19).

### Statement 12: Established cardiovascular disease risk factors should be actively investigated and controlled in patients with IBD

- Consensus reached for 100%. Vote: fully agree 73%, mostly agree 27%.
- Evidence level 4.

Patients with IBD should be screened for CVD risk factors, as atherosclerosis is responsible for most arterial thrombotic events. Several factors are associated with an increased risk of atherosclerotic plaques in multiple arterial beds (coronary, cerebrovascular, peripheral, mesenteric). Traditional CVD risk factors include hypertension, diabetes, hyperlipidaemia, smoking and obesity. Additionally, risk-enhancing factors can further modify the CVD risk and can influence the need for primary prevention therapies. They include family history of premature atherosclerotic CVD, familial hypercholesterolaemia, metabolic syndrome, chronic kidney disease, chronic inflammatory conditions, history of premature menopause or pregnancy-associated conditions, ethnicities or races at high risk (such as South Asian ancestry), lipid abnormalities (including hypertriglyceridaemia or elevated lipoprotein(a) or apoB), and elevated CRP level^98^. Other biological risk factors for CVD include hyperhomocysteinaemia^99^ owing to vitamin deficiencies in relation to altered absorption^100^ and antiphospholipid antibodies either primary or mediated by a concomitant autoimmune disease (for example, systemic lupus erythematosus), or chronic inflammation^36^. These biological risk factors increase the risk of developing both arterial and venous thromboses.

CVD risk factors are not universally over-represented in patients with IBD (see Statement 7). Additionally, treatments (particularly steroids) prescribed for flares as well as the inflammatory nature of IBD itself can also increase CVD risk^101,102^.

Several models and calculators for CVD risk assessment that take into account these risk factors have been developed^98,103–105^, but these are beyond the scope of this paper. However, these tools fail to reliably identify patients <40 years of age at high risk^83,104^. Patients with IBD should be advised not to smoke and to follow a healthy lifestyle to avoid obesity. Smoking cessation should be strongly advised: smoking is a known risk factor for arterial and venous events per se^106,107^, and can also increase the risk of these events further through disease activity, given that smoking is associated with more aggressive disease, especially in Crohn’s disease^108^. Smoking, hypertension and hypercholesterolaemia should be systematically screened for according to age-specific guidelines for the general population. Owing to a higher risk of CVD, selected patients with IBD (such as those older than 40 years or younger patients with known risk factors) should be referred to general practitioner and/or cardiologist or vascular medicine specialist for screening for and control of CVD risk factors.

Regarding antiplatelet therapy, it is recommended that all patients with a history of an arterial atherosclerosis-related thrombosis event should be prescribed long-term low-dose aspirin for secondary prevention^109^. Low-dose aspirin in primary prevention has been extensively studied and is still controversial even in high-risk populations due to an unfavourable benefit–risk ratio^110–113^. This issue is particularly true in older patients >70 years of age who have a high risk of bleeding^114^. Since the increasing prescription of statins during the past few decades to reduce the risk of a first CVD event due to plaque rupture, the beneficial effect of aspirin has been challenged^114^. Thus, in primary prevention, low-dose aspirin should be discouraged in older patients >70 years of age and can be considered in young patients with a high CVD risk who still have uncontrolled CVD risk factors despite conventional treatments (such as statins or antihypertensive drugs)^98^. In patients with IBD, the use of aspirin seems to be safe and is not related to worse disease outcomes. In a retrospective study in 2021 that included 764 patients with IBD, aspirin use was not associated with IBD-related hospitalization, corticosteroid use, or IBD-related surgery^115^.

## Risk of drug-related thrombosis in patients with IBD

### Statement 13: There is limited evidence regarding the effect on the risk of VTE with the use of 5-ASA, thiopurines or methotrexate in patients with IBD

- Consensus reached for 100%. Vote: fully agree 47%, mostly agree 53%.
- Evidence level 3.

There is a paucity of evidence regarding the potential effect of 5-aminosalicylic acid (5-ASA) compounds on the risk of VTE. A small study showed that, independent of diagnosis or disease activity, spontaneous ex-vivo platelet activation was 50% lower in patients with IBD taking 5-ASA orally than in those not on such treatment (*P* < 0.05)^116^. In vitro, 5-ASA significantly reduced both spontaneous (*P* < 0.03) and thrombin-induced platelet activation (*P* < 0.02)^116^. These observations could lead to the assumption that 5-ASA could theoretically reduce the risk of VTE. On the other hand, a previous small study in six patients with IBD (four with ulcerative colitis, and two with Crohn’s disease) treated with 5-ASA showed no changes in platelet aggregation or fibrinolytic activity^117^. These are small limited studies, and clinical evidence regarding the risk of VTE in patients with IBD on 5-ASA is lacking. However, data from RCTs investigating the use of mesalazine in patients with ulcerative colitis have not shown any safety signal towards an increased risk of VTE^118^.

Indirect data suggest a potential antithrombotic effect from thiopurines. One study showed that azathioprine inhibits in vitro platelet aggregation^119^. Additionally, another study showed that patients with IBD on thiopurines had fewer leukocyte–platelet aggregates than patients who were not on thiopurines^120^. As with 5-ASA, there is paucity of clinical data showing a reduced risk of thrombotic events with the use of thiopurines in patients with IBD, and accumulated evidence does not show an increased risk of VTE with immunomodulators. For example, in a prospective cohort study that included 245 patients with IBD exposed to thiopurines with a median follow-up of 32 months (range 0.2–75 months), no VTE event was reported^121^.

Methotrexate can increase the levels of homocysteine in the absence of folate supplementation, which may potentially increase the risk of VTE^122,123^. This could be particularly relevant given that patients with IBD have an increased risk of hyperhomocysteinaemia^124^. A meta-analysis from 2011 summarized data from 28 studies and found that risk of hyperhomocysteinaemia was significantly higher in patients with IBD when compared with controls (OR 4.65, 95% CI 3.04–7.09)^124^. However, its effect on the risk of thrombosis in patients with IBD is less clear, as the odds of having hyperhomocysteinaemia was not increased among patients who experienced thrombotic complications (OR 1.97, 95% CI 0.83–4.67)^124^. In patients with rheumatoid arthritis taking methotrexate, folate supplementation was associated with normalization of homocysteine levels^123^. Folate supplementation should be advised to avoid hyperhomocysteinaemia in patients with IBD. A retrospective cohort study of 782 patients with IBD starting thiopurines or methotrexate found that 27% discontinued therapy owing to adverse events (40% on methotrexate versus 19% on thiopurines, *P* < 0.001), but none discontinued therapy owing to thrombotic events^125^.

### Statement 14: 5-ASA is associated with a reduced risk of ischaemic heart disease in patients with IBD, especially when given in the long term

- Consensus reached for 100%. Vote: fully agree 36%, mostly agree 64%.
- Evidence level 2−.

A nationwide population-based cohort study from Denmark by Rungoe et al. included 4,570,820 individuals, of whom 28,833 were diagnosed with IBD (7,521 with Crohn’s disease, 19,990 with ulcerative colitis)^126^. During up to 13 years of follow-up, 245,019 incident cases of IHD were identified, of which 1,175 occurred among patients with IBD. ﻿Patients with IBD had a significantly increased risk of IHD (incidence rate ratio (IRR) 1.59, 95% CI 1.50–1.69) compared with those without IBD^126^. Importantly, 5-ASA users had a lower risk of IHD than those who did not use 5-ASA (IRR 1.16 versus 1.36; *P* = 0.02), even after adjustment for corticosteroid use as a proxy for disease severity. In long-term users of 5-ASA ﻿(defined as those with three or more redeemed prescriptions), the risk was even lower (IRR 1.08, 95% CI 0.98–1.19) and was non-significant compared with individuals without IBD^126^. A cardioprotective effect of 5-ASA could be explained by salicylic acid being the main active fraction of 5-ASA as well as of aspirin. However, a causal relationship cannot be fully established from these results, given that population-based studies do not allow adequate discrimination between 5-ASA use, disease activity and IHD. Further studies are needed to confirm the cardioprotective effect of 5-ASA, and currently there is insufficient evidence to recommend the use of 5-ASA in patients with IBD solely to prevent arterial events.

### Statement 15: Steroids are associated with an increased risk of venous and arterial thrombotic events in patients with IBD

- Consensus reached for 100%. Vote: fully agree 31%, mostly agree 69%.
- Evidence level 2++.

In a retrospective study, Higgins et al. included 15,100 patients with IBD and identified 335 VTE events during the period 2003–2009 (ref.^127^). ﻿The absolute rates of VTE ﻿within 12 months after an index prescription were 2.25% (296 of 13,165), 0.44% (2 of 452), and 2.49% (37 of 1,483) for patients exposed to corticosteroid only, biologic agent only, and combination of corticosteroid plus a biologic agent, respectively. ﻿When compared with corticosteroid monotherapy, monotherapy with a biologic agent was associated with an adjusted OR of 0.21 (95% CI 0.05–0.87) for VTE, whereas for combinations of corticosteroids plus a biologic agent the adjusted OR was ﻿1.01 (95% CI 0.71–1.45)^127^. Importantly, ﻿after controlling for covariates, the use of high-dose corticosteroids was associated with an OR of 3.31 (95% CI, 2.50–4.37) compared with low-dose corticosteroids, indicating a dose–response effect on the risk of VTE. The authors also evaluated the risk of VTE over time; they found no additional VTE events after 2 months in patients receiving monotherapy with a biologic agent, whereas in those receiving corticosteroids (monotherapy or combined with a biologic agent), VTE events continued to occur during up to 12 months of follow-up^127^.

In a retrospective cohort study from the ﻿Veterans Health Administration including ﻿30,456 patients with IBD^128^, the incidence rate of VTE increased after the diagnosis of IBD, although this increase was more pronounced in patients exposed to corticosteroids than in patients not exposed (3.1 to 9.0 per 1,000 person-years versus 2.1 to 4.9 per 1,000 person-years). In patients exposed to corticosteroids the rate of VTE increased more than fivefold (16.9 per 1,000 person-years) in the year after corticosteroid exposure compared with the year prior to diagnosis^128^.

In a meta-analysis published in 2018, six studies were included assessing the risk of VTE in patients with IBD treated or not with systemic corticosteroids^129^. A total of 40,083 patients with IBD were analysed and 2,861 (7.13%) VTE events were identified. There was a significantly higher rate of VTE in patients treated with corticosteroids than in patients without steroid medication (OR 2.20, 95% CI 1.70–2.86; *P* < 0.001)^129^.

Systemic corticosteroids seem to be also associated with an increased risk of arterial thrombotic events, as seen in other immune-mediated diseases and in the general population^130,131^. In a nested case–control study, Andersohn et al.^132^ found that patients with Crohn’s disease who had a CVA were more likely to have received a corticosteroid prescription in the preceding 3 months than patients who did not have a CVA (adjusted OR 1.71, 95% CI ﻿1.34–2.19). In the aforementioned population-based study by Rungoe et al., ﻿patients who required oral corticosteroids had a significantly higher risk of IHD (IRR 1.37, 95% CI 1.25–1.50) than patients who had never received oral corticosteroids (IRR 1.23, 95% CI 1.12–1.36; *P* < 0.01)^126^. However, it should be noted that corticosteroid use can be regarded as a proxy for disease activity, and the latter is a known risk factor for arterial events (see Statement 7); hence a causal relationship cannot be fully established.

### Statement 16: Anti-TNF agents can be associated with a decreased risk of VTE in patients with IBD

- Consensus reached for 100%. Vote: fully agree 41%, mostly agree 59%.
- Evidence level 2+.

TNF has been implicated in accelerated thrombus formation and activation of coagulation^6^, so anti-TNF agents might have a potential protective effect against VTE, besides their intrinsic efficacy in reducing disease activity.

A prospective study included 103 patients with IBD starting infliximab and 113 healthy individuals as controls found that patients with IBD have ﻿increased concentrations of active inhibitors of the fibrinolytic system, which might be involved in the increased risk of thrombotic events. Interestingly, patients who responded to infliximab therapy had a normalization of their clot lysis profile^133^. These results were confirmed in a more recent study supporting the notion that anti-TNF agents reduce the risk of VTE^134^.

In a retrospective cohort study of 547 hospitalized patients with IBD, systemic corticosteroid use (OR 4.62, 95% CI 1.98–10.80) was associated with an increased risk of VTE, whereas anti-TNF agents were associated with a reduced risk (OR 0.20, 95% CI 0.04–0.99)^135^. A cohort study ﻿that included ﻿5,173 patients with IBD, evaluated the risk of VTE in patients exposed to anti-TNF agents and in those exposed to non-biologic drugs (thiopurines, methotrexate or ciclosporin)^136^. The authors did not find a statistically significant difference between the groups in the overall population (adjusted HR 0.78, 95% CI 0.60–1.02), although in patients with Crohn’s disease (HR 0.62, 95% CI 0.44–0.86) and those younger than 45 years (﻿HR 0.55, 95% CI 0.34–0.87) a protective effect of anti-TNF agents reached statistical significance^136^. A meta-analysis by Sarlos et al. included three studies that compared the risk of VTE in patients with IBD treated with anti-TNF agents versus systemic corticosteroids, including 18,435 patients with 399 (2.2%) VTE events. A significantly lower rate of VTE events was seen in patients with IBD treated with anti-TNF agents than those treated with steroids (OR 0.27, 95% CI 0.11–0.67)^129^.

In a prospective cohort study evaluating the long-term safety outcomes of infliximab treatment, a higher incidence of VTE was seen in patients on anti-TNF agents (0.16 events per 100 patient-years) than in patients on other treatments, such as thiopurines or methotrexate (0.10 events per 100 person-years)^137^. However, these estimates were not adjusted for disease severity and other covariates. There are no consistent safety signals of an increased risk of VTE in patients with IBD treated with vedolizumab or ustekinumab. A study by Cross et al. found that patients with Crohn’s disease exposed to vedolizumab had an increased incidence of PE (IRR 3.01, 95% CI 1.11–8.18) and DVT (IRR 2.67, 95% CI 1.32–5.41) compared with those exposed to anti-TNF agents^138^. These findings should be interpreted with caution given the small absolute number of VTE events in patients on vedolizumab (*n* = 12) and also the fact that patients exposed to vedolizumab had higher corticosteroid use (78.8% versus 48.9%). Additionally, RCTs and large real-world studies have not reported VTE events^139–141^. There is a paucity of studies evaluating the risk of VTE with the use of ustekinumab in the IBD population. In the Psoriasis Longitudinal Assessment and Registry (PSOLAR) study, in which 12,093 patients with psoriasis were enrolled (40,388 patient-years of follow-up), there were no reports of VTE in patients exposed to ustekinumab (40,388 patient-years of follow-up)^142^.

### Statement 17: Anti-TNF agents can be associated with a reduced risk of arterial events in patients with IBD

- Consensus reached for 94%. Vote: fully agree 29%, mostly agree 65%.
- Evidence level 2+.

In a retrospective cohort study, Lewis et al.^143^ found that among patients with Crohn’s disease (7,694 with prolonged corticosteroid use and 1,879 with anti-TNF agent use) the risk of death was statistically significantly lower in patients treated with anti-TNF therapy than in patients on corticosteroids (OR 0.78, 95% CI 0.65–0.93) and also anti-TNF therapy was associated with lower rates of major adverse cardiovascular events (MACE) (OR 0.68, 95% CI 0.55–0.85).

In a 2020 nationwide population-based cohort study from France including 177,827 patients with IBD, ﻿Kirchgesner et al. analysed the effect of thiopurines and anti-TNF agents on the risk of acute arterial events (﻿IHD, CVA and PAD)^144^. Patients exposed to anti-TNF agents had a lower risk of acute arterial events than patients without exposure to anti-TNF agents or thiopurines (HR 0.79, 95% CI 0.66–0.95). The magnitude of the risk reduction was highest in men with Crohn’s disease exposed to anti-TNF agents (HR 0.54, 95% CI 0.40–0.72). Notably, in patients exposed to thiopurines the risk was not significantly changed. A meta-analysis that included 24 studies found that exposure to biologic agents in patients with an IMID was associated with a 30% lower odds of cardiovascular events (OR 0.70, 95% CI 0.59–0.82)^145^.

There is paucity of data regarding the risk of arterial events with the use of newer biologic agents (that is, vedolizumab and ustekinumab) in patients with IBD. Data from clinical trials and post-marketing safety reports do not show any safety signal towards an increased risk of venous or arterial events in patients with IBD^146,147^. Regarding ustekinumab, a meta-analysis evaluated the risk of MACE in patients with psoriasis receiving biologic agents (eight RCTs involving 3,862 patients) and found no significant increased risk of MACE in patients exposed to ustekinumab (OR 4.48, 95% CI 0.24–84.77)^148^. However, a 2020 case–time–control study suggested that the initiation of treatment with ustekinumab is associated with an increased risk of severe arterial events (acute coronary syndrome or stroke) in patients with a high baseline cardiovascular risk^149^. The majority of patients had psoriasis (91%) with only 6% having Crohn’s disease^149^. This observation needs to be confirmed in further studies, and its relevance in patients with IBD remains unclear.

### Statement 18: Tofacitinib can be associated with a dose-dependent increased risk of VTE in patients with rheumatoid arthritis with risk factors for VTE. According to available evidence, no increase in the risk of VTE has been observed in the overall ulcerative colitis population treated with tofacitinib

- Consensus reached for 100%. Vote: fully agree 50%, mostly agree 50%.
- Evidence level 1+.

In a recently completed, open-label, post-marketing study, the safety of tofacitinib versus an anti-TNF agent was evaluated. Patients with moderate to severe rheumatoid arthritis refractory to methotrexate, older than 50 years, and with at least one cardiovascular risk factor were enrolled (ORAL Surveillance Study; study A3921133, NCT02092467). Analysis in February 2019 showed statistically and clinically important differences in the occurrence of PE (HR 5.96, 95% CI 1.75–20.33) and mortality (HR 3.28, 95% CI 1.55–6.95) between patients receiving tofacitinib 10 mg twice daily (BID) and those receiving the anti-TNF agent. Importantly, the incidence rates of PE and mortality among patients receiving tofacitinib 10 mg BID were higher in those with background risk factors for VTE than in patients without them. Based on these results, both the FDA and EMA have released a warning^150,151^, and the FDA has limited the use of tofacitinib to patients with ulcerative colitis that are refractory or intolerant to treatment with anti-TNF agents. The mechanism involved in the potential increased risk of VTE associated with tofacitinib is poorly understood.

In a large cohort study by Desai et al. in 50,865 patients with rheumatoid arthritis starting treatment with tofacitinib or anti-TNF agents, a numerically, but statistically non-significantly, higher risk of VTE was seen in patients receiving tofacitinib (unadjusted HR ﻿1.42, 95% CI 0.84–2.40)^152^. Notably, a greater proportion of patients starting tofacitinib were receiving more than three non-biologic disease-modifying antirheumatic drugs and corticosteroids at baseline, probably indicating more severe disease or a longer duration of the active systemic inflammation^152^. A meta-analysis published in 2020 that ﻿included ten controlled studies and ﻿5,143 patients exposed to JAK inhibitors did not find significant differences in the risk of VTE with the use of JAK inhibitors in patients with an IMID (﻿RR 0.90, 95% CI 0.32–2.54)^153^.

A post hoc analysis evaluated the occurrence DVT and PE in the tofacitinib development programme in ulcerative colitis, from the induction and maintenance studies and from the open-label extension (OLE) study^154^. In this analysis, one patient developed DVT and four patients had PE during treatment with tofacitinib, out of 1,157 patients in the overall cohort. All VTE events in patients receiving tofacitinib at the time of the event occurred during the OLE study, after at least 7 months of treatment (tofacitinib exposure range 216–1,149 days) and in patients receiving 10 mg BID. All these patients had at least one VTE risk factor at the same time (for example, obesity, hormone-replacement therapy, prior history of VTE)^154^. Importantly, during the induction and maintenance studies, four patients developed VTE events; all were receiving placebo at the time of event, and none of the tofacitinib-exposed patients developed VTE events during the induction and maintenance studies of the OCTAVE programme. In the overall cohort, the incidence rate of PE in all patients exposed to tofacitinib (2,403.6 patient-years of exposure) was 0.16 per 100 patient-years (95% CI ﻿0.04–0.41), whereas in patients exposed to the predominant dose of tofacitinib 10 mg BID (1,808.1 patient-years of exposure) it was 0.21 per 100 patient-years (95% CI ﻿0.06–0.55). These incidence rates for VTE events with tofacitinib were comparable with those previously reported for patients with ulcerative colitis^155^. Given these results, the risk of VTE does not seem to be increased in patients with ulcerative colitis exposed to tofacitinib. However, further studies in a population of patients with ulcerative colitis enriched with VTE risk factors are needed to fully determine whether this safety signal seen in patients with rheumatoid arthritis is also applicable to those with ulcerative colitis.

In a real-world cohort of 260 patients with ulcerative colitis exposed to tofacitinib with a median follow-up time of 6 months (interquartile range 2.7–11.5 months), VTE was identified in two patients, giving an IRR for VTE of 1.32 per 100 patient-years of follow-up (95% CI 0.33–5.28). Both patients were on tofacitinib 10 mg BID at the time of the event and had provoking risk factors for VTE^156^.

Given that VTE has been highlighted as a potential risk during treatment with tofacitinib, physicians should acknowledge this possibility and individualize management by screening for risk factors for VTE (Statement 2) prior to and during treatment with tofacitinib in patients with ulcerative colitis. Physicians should also aim for the lowest effective dose of tofacitinib. Tofacitinib 10 mg BID should be used as induction therapy for up to 16 weeks. Tofacitinib 5 mg BID should be the preferred maintenance dose. In patients with insufficient response to the maintenance dose, a dose increase to 10 mg BID could be considered in patients without known risk factors for VTE and without therapeutic alternatives.

### Statement 19: Tofacitinib is not associated with an increased risk of major adverse cardiovascular events in patients with ulcerative colitis

- Consensus reached for 100%. Vote: fully agree 50%, mostly agree 50%.
- Evidence level 1−.

Tofacitinib has been associated with alterations in the serum lipids profile and, given that hypercholesterolaemia is a known risk factor for cardiovascular events in general population, the possible occurrence of MACE with the use tofacitinib has long been a concern. However, changes seen in cholesterol levels are small and transient, with the LDL to HDL ratio usually stable. Additionally, these changes have been shown to be reversible with statin treatment in patients with rheumatoid arthritis^157^.

A post hoc analysis of the tofacitinib development programme in ulcerative colitis found an incidence rate per 100 years of exposure of 0.24 (95% CI 0.07–0.62; median treatment duration 514 days; exposure 1,612.8 patient-years)^158^. This finding is in line with the incidence rates of MACE seen with the use of anti-TNF agents in patients with ulcerative colitis (incidence rate ﻿0.51, 95% CI 0.31–0.79)^159^. MACEs were reported in four patients exposed to tofacitinib; three of these patients had four or more traditional cardiovascular risk factors, including hyperlipidaemia, hypertension, diabetes mellitus, history of smoking, and/or family history of CAD^160^. However, the majority of patients in the cohort did not have cardiovascular risk factors at baseline, with only 6.1% of patients taking lipid-lowering medications at baseline^158^.

In a meta-analysis by Olivera et al., no significant increased risk of MACE was seen in patients with IBD exposed to JAK inhibitors, as well as in the risk of a myriad of IMIDs (RR 1.07, 95% CI 0.56–2.01)^153^. Another meta-analysis by Xie et al.^161^ including 29 RCTs evaluating tofacitinib in patients with IMIDs found no significant increase in the risk of all cardiovascular events (OR 1.07, 95% CI 0.49–2.34), MACE (OR 1.54, 95% CI 0.42–5.59), or all-cause mortality (OR 1.13, 95% CI 0.26–4.95).

## Conclusions

The ultimate goal of this consensus meeting was to give recommendations to improve the quality of care of patients with IBD and prevent potentially life-threatening complications of thrombosis. This consensus meeting led to the development of a series of statements (Box 1) supported by available evidence regarding the background risk of thrombotic events in patients with IBD, as well as how this risk is modulated by commonly used drug therapies in IBD (that is, 5-ASA, thiopurines, methotrexate, steroids, biologic agents and tofacitinib). Additionally, whenever appropriate consensus recommendations for the prevention of venous, arterial and drug-related thrombosis are made (Table 2). Treatment strategies of established thrombotic events in patients with IBD were beyond the scope of this consensus, and physicians should refer to available guidelines^32^.

Further evidence is needed regarding the drug-related risk of thrombosis with newer therapies in the IBD population, specifically the risk of MACE with ustekinumab and the risk of VTE with tofacitinib and other JAK inhibitors. Real-world studies as well as controlled studies specifically designed to address this important issue are warranted. Development of specific risk assessment tools for thrombotic complications in patients with IBD are needed, as they might influence management in some clinical scenarios (such as thromboprophylaxis during ambulatory flares).

## Supplementary Information

Supplementary Information
