# Supplementary material for: International consensus on the prevention of venous and arterial thrombotic events in patients with inflammatory bowel disease
Source: Nat Rev Gastroenterol Hepatol. 2021 Aug 27;18(12):857–73. doi: 10.1038/s41575-021-00492-8 (PMC8395387; doi:10.1038/s41575-021-00492-8)
Supplement: Supplementary file 1 — Supplementary Information [file 41575_2021_492_MOESM1_ESM.pdf]

nature portfolio

https://doi.org/10.1038/s41575-021-00492-8

Supplementary information

International consensus on the prevention of venous and arterial thrombotic events in patients with inflammatory bowel disease

In the format provided by the authors and unedited

Supplementary information

Supplementary Table 1 | MEDLINE Search strategies

1. ("Inflammatory bowel diseases" OR "inflammatory bowel disease") OR (Crohn's OR Crohn) OR "ulcerative colitis" OR colitis
2. "Venous thromboembolism" OR "pulmonary embolism" OR ("deep vein thrombosis" OR "venous thrombosis")
3. Thromboprophylaxis OR "thrombosis prophylaxis" OR prevention
4. Thrombophilia
5. Heparin OR "low molecular weight heparin" OR enoxaparin OR fondaparinux OR ("direct oral anticoagulant" OR rivaroxaban OR apixaban OR dabigatran)
6. ("major adverse cardiovascular event" OR MACE) OR "arterial event" OR "arterial thrombosis" OR "ischemic heart disease" OR "myocardial infarction" OR "cardiovascular death" OR ("cerebrovascular disease" OR "cerebrovascular accident" OR stroke OR "transient ischemic attack") OR "mesenteric ischemia" OR ("peripheral artery disease" OR "limb ischemia")
7. (Steroid OR steroids OR corticosteroids) OR (5-ASA OR "5-amynosalicilate" OR mesalazine OR mesalamine OR sulfasalazine) OR (thiopurine OR azathioprine OR mercaptopurine) OR methotrexate
8. ("anti-TNF" OR "anti-tumor necrosis factor" OR "anti-tumour necrosis factor" OR "TNF-antagonist") OR vedolizumab OR ustekinumab OR (tofacitinib OR "JAK inhibitor" OR "Janus kinase inhibitor")

Supplementary Table 2 | SIGN grading system

**Levels of evidence**	
1++	High quality meta-analyses, systematic reviews of RCTs, or RCTs with a very low risk of bias
1+	Well-conducted meta-analyses, systematic reviews, or RCTs with a low risk of bias
1-	Meta-analyses, systematic reviews, or RCTs with a high risk of bias
2++	High quality systematic reviews of case control or cohort studies<br>High quality case control or cohort studies with a very low risk of confounding bias and a high probability that the relationship is causal
2+	Well-conducted case control or cohort studies with a low risk of confounding or bias and a moderate probability that the relationship is causal
2-	Case control or cohort studies with a high risk of confounding or bias and a significant risk that the relationship is not causal
3	Non-analytic studies (e.g. case reports, case series)
4	Expert opinion

Grades of recommendations	
A	At least one meta-analysis, systematic review, or RCT rated as 1++, and directly applicable to the target population; or<br>A body of evidence consisting principally of studies rated as 1+, directly applicable to the target population, and demonstrating overall consistency of results
B	A body of evidence including studies rated as 2++, directly applicable to the target population, and demonstrating overall consistency of results; or<br>Extrapolated evidence from studies rated as 1++ or 1+
C	A body of evidence including studies rated as 2+, directly applicable to the target population and demonstrating overall consistency of results; or<br>Extrapolated evidence from studies rated as 2++
D	Evidence level 3 or 4; or Extrapolated evidence from studies rated as 2+

RCT, randomized controlled trial.

Supplementary Table 3 | Excluded statements

1.	Patients receiving methotrexate, should receive concomitant folate supplementation, in order to reduce hyperhomocysteinemia.
2.	Anti-TNF agents should be considered over steroids to treat flare-ups in IBD patients with known risk factors for VTE.
3.	There is insufficient evidence regarding the risk of thrombotic events with vedolizumab.
4.	There is insufficient evidence regarding the risk of thrombotic events with ustekinumab in IBD, although ustekinumab does not influence the risk of MACE in psoriasis.
5.	Physicians should aim to the lowest effective dose. Tofacitinib 10 mg BID should be used as induction therapy for up to 16 weeks. Tofacitinib 5 mg BID should be used as maintenance dose. In patients with insufficient response to maintenance dose, dose increase to 10 mg BID could be considered in patients without known risk factors of VTE, and without therapeutic alternatives.
6.	JAK inhibitors are associated with changes in the lipid profile, particularly of low-density lipoprotein concentrations; these changes should be adequately investigated and treatment with statins should be considered.

BID, twice daily; JAK, Janus kinase; MACE, major cardiovascular event; VTE, venous thromboembolism.
